# Metastatic non-functional paraganglioma to the lung

**DOI:** 10.1186/s13019-020-01113-2

**Published:** 2020-05-11

**Authors:** Mohamad K. Abou Chaar, Aseel Khanfer, Nidal M. Almasri, Mohammad Abu Shattal, Abdellatif O. Alibraheem, Obada Al-Qudah

**Affiliations:** 1 Department of Surgery, King Hussein Cancer Center, Amman, Jordan; 2 Department of Pathology and Laboratory Medicine, King Hussein Cancer Center, Amman, Jordan; 3 Department of Radiology, King Hussein Cancer Center, Amman, Jordan; 4 Department of Thoracic Oncology, King Hussein Cancer Center, Amman, Jordan

**Keywords:** Paraganglioma, pulmonary metastasis, Single port VATS

## Abstract

**Introduction:**

Paragangliomas are rare endocrine tumors that arise from the extra-adrenal autonomic paraganglia and sympathetic paragangliomas usually secret catecholamines and are located in the sympathetic paravertebral ganglia of thorax, abdomen, and pelvis. In contrast, most parasympathetic paragangliomas are nonfunctional and located along the glossopharyngeal and vagal nerves in the neck and at the base of the skull. Such neoplasms, although rare, are clinically important because they may recur after surgical resection and 10% of them give rise to metastases causing death with the lymphatic nodes, bones, liver, and lungs being the most common locations.

**Case presentation:**

We present a case of a 26-year-old male patient that was diagnosed with paraganglioma of the right-frontal lobe infiltrating the falx and frontal bone which was diagnosed after suffering from a headache and abnormal vision. On initial work-up he was found to have right pulmonary nodules that increased in size after follow up and other nodules appeared in the contralateral lung. He underwent subtotal resection of the brain tumor and complete resection of the bilateral pulmonary nodules.

**Conclusion:**

To our knowledge, paraganglioma is considered to be a rare entity in the central nervous system with very few cases being reported in the supratentorial region and no cases were reported of metastatic such paraganglioma to the lung.

## Introduction

Paragangliomas (PGLs) are rare chromaffin cell tumors, with the first case described by Dr. Felix Fränkel in 1886, which can often be cured by surgical resection. The diagnosis of PGL remains a challenge, because patients do not present with characteristic signs and symptoms. In recent years, it was noted that metastatic disease in PGL was more frequent in patients presenting with extra-adrenal PGL, with a PGL exceeding a size of 5 cm and/or carrying an SDHB germline mutation [1]. In up to 10 % of patients, metastases are already present at diagnosis of PGL [2]. However, once metastatses are present, treatment options are limited to one or a combination of a] excision or ablation, b] targeted pharmacotherapy, c] chemotherapy [3–5] Survival of patients with metastatic PGL is variable with overall 5 year survival is 35–60 %, [6]. Here we present a case of right frontal brain non-functional paraganglioma that was associated with bilateral lung nodules in which the patient underwent staged metastasectomies of the pulmonary and pleural nodules via single-port video-assisted thoracoscopic surgery (VATS) after debulking of the primary tumor.

## Case presentation

**Fig. 1:**
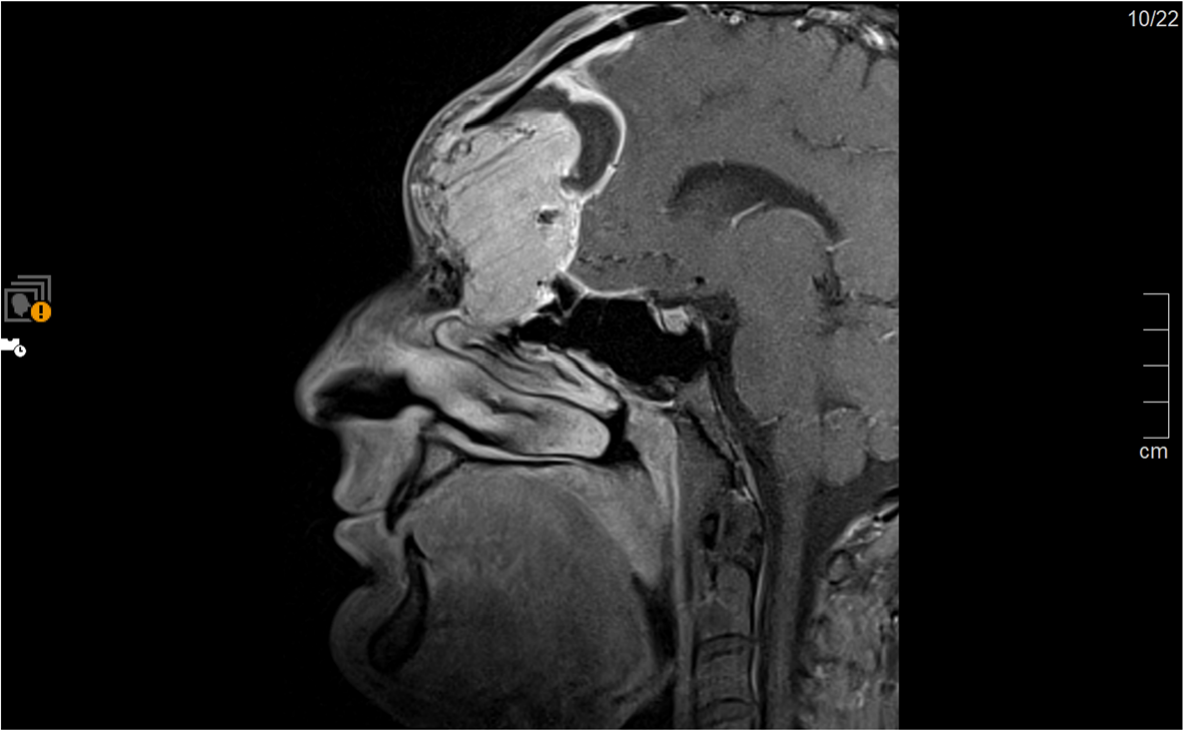
Sagittal view. Post-contrast T1 weighted image. Showing bifrontal mass destructing the frontal bone with right intraoribital extension and bilateral front and ethmoidal sinuses extension

A 26-year-old male patient, who is known to have hepatitis B, presented to his primary care physician in December, 2015 with a small mass on his forehead of one month duration, painless, associated with sudden onset of diplopia, mainly when looking towards the left, not associated with any other symptom. He was previously diagnosed with Ewing sarcoma of the 8^th^ right rib at the age of eleven years for which he underwent surgical resection, followed by 7 cycles of Vincristine, Actinomycin, and Ifosfamide (last cycle was on 13^th^ of May 2004) and 30 sessions of radiotherapy, and has been free of disease until the date of presentation. Complete neurological examination and hormonal assessment were normal apart from right eye ptosis and movement restriction towards both sides. Magnetic resonance imaging (MRI) of brain was done which showed an expansile heterogeneous bony extra-axial mass lesion seen at the right frontal region, this mass did not show diffusion restriction, however, vivid heterogeneous enhancement with central necrosis seen after contrast media injection. Multiple T1 hyperintense foci which may represent calcification/hemorrhage , it is seen to invade the right frontal and right ethmoidal sinuses as well as the right orbital roof , it measured 6.2cm(AP)x5.2cm(trans)x6.2 cm (c.c), causing midline shift to the left side by 1.2cm without any surrounding edema or hydrocephalus . Brain magnetic resonance angiogram (MRA) showed that the anterior cerebral artery (ACA) appeared to be displaced to the left side. Whole body computed tomography (CT) scan was done which had no significant changes apart from two pulmonary nodules, one seen at the left upper lobe measuring 8mmx4.5mm and one seen at the right lower lobe measuring 4.5mm in diameter. He underwent multiple craniotomy trials that ended with incomplete excision of tumor due to massive bleeding and a definitive diagnosis of paraganglioma was achieved followed by brain MRI which showed incompletely excised tumor extending to the nasal cavity, and frontal and ethmoidal sinuses [Fig. 1].

**Fig. 2:**
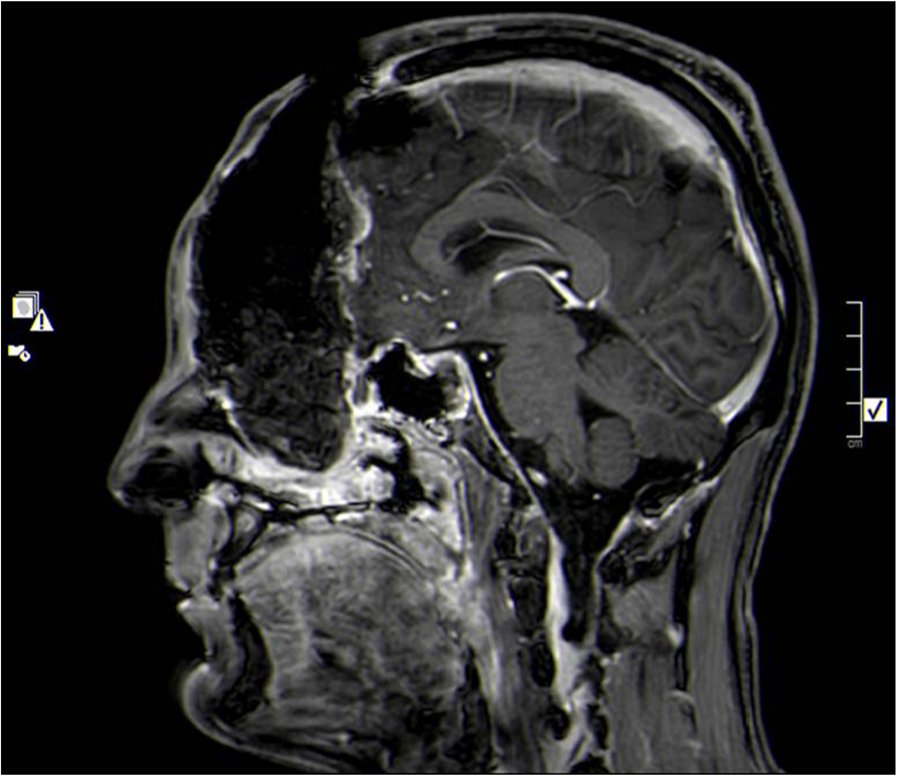
Brain MRI. Sagittal view. Post-contrast T1 weighted image. Showing post-operative cavity without gross residual mass

In July of 2016, and after referral to King Hussein Cancer Center (KHCC), histopathology confirmed the diagnosis of paraganglioma followed by a new whole body CT scan showed subpleural nodules seen in the lingula, measures 1cm x 1 cm, and few subpleural nodules seen in the right as well, largest seen in right lower lobe, measured around 0.5cmx0.5cm. A Multidisciplinary team sat down with the patient and explained the surgical management of the metastatic disease and explained the staged procedures that will take place, starting with excision of the primary tumor and then targeting the pulmonary nodules, all outcomes were explained and the patient agreed to undergo four vessel cerebral angiogram for pre-operative assessment followed by bifrontal craniotomy, in January, 2017, which resulted in major debulking of the mass and complicated by profuse bleeding [Fig. 2]. Post-operative MRI showed small residual tumor in the right maxillary sinus, after which he received 28 session of 50.4 Gy / 28 Fx via Intensity Modulated Radiation Therapy (IMRT).

**Fig. 3:**
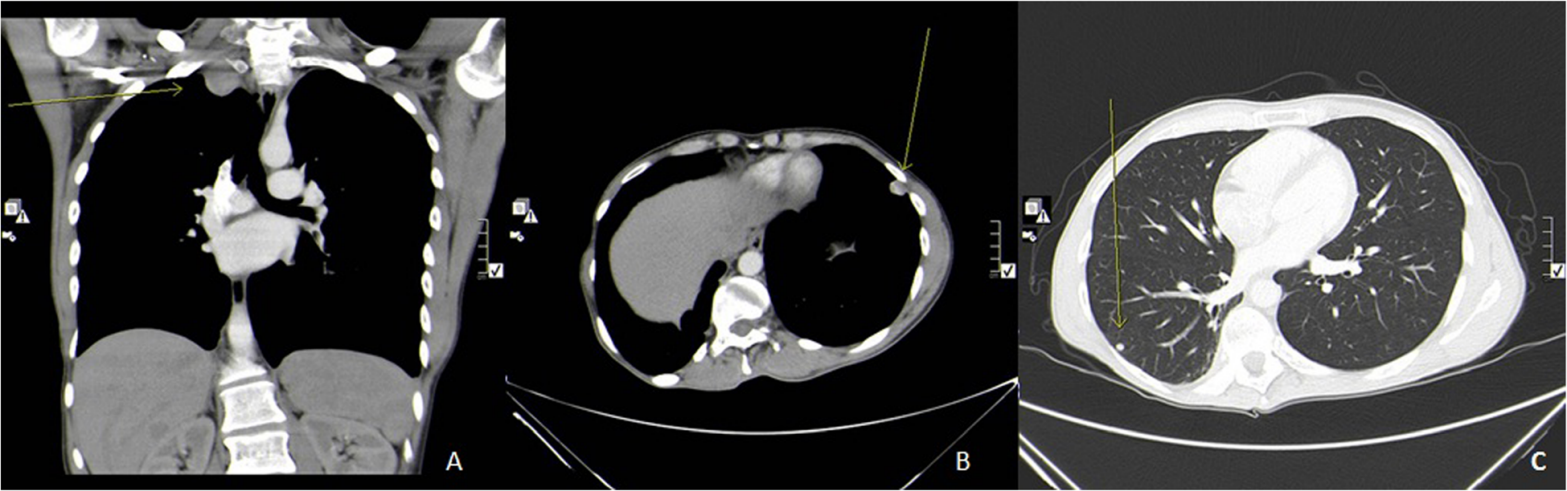
Contrast enhanced chest CT scan. **a**. Coronal view, mediastinal window. **b**. Sagittal view, mediastinal window. **c**. Sagittal view, pulmonary window. Showing three subpleural pulmonary metastasis indicated by yellow arrow

**Fig. 4:**
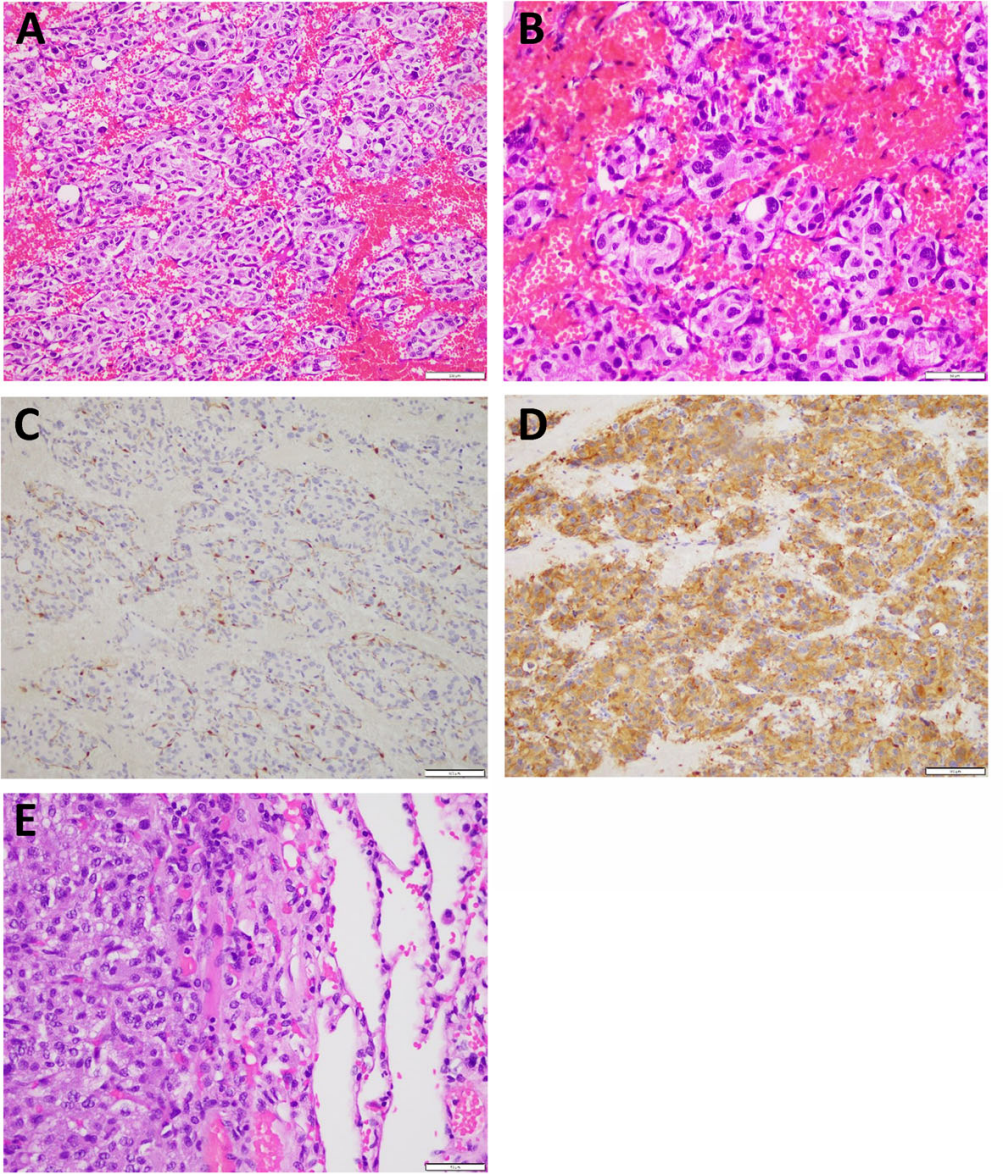
Microscopic pictures representative of the patient’s tumor. **a**: Hematoxylin and eosin stain (H&E) 20X of the frontal lobe tumor showing organoid nests of tumor cells separated by vascular septa. The tumor cells are cuboidal and have granular eosinophilic cytoplasm. **b**: Higher power view (H&E, 40X) illustrating the tumor nests surrounded by sustentacular cells; hyperchromasia and nuclear pleomorphism are evident. One atypical mitosis is seen at the center of the picture. **c**: Immunostain for S-100 protein showing positive brown staining that highlights the sustentacular cells (40X). **d**: Imunostain for synaptophysin showing brown cytoplasmic staining in the tumor cells (40X). **e**: Metastatic paranglioma seen from this lung lesion. Normal pulmonary alveoli are seen at the right side of the picture, and paraganglioma, similar to the primary frontal lobe mass, is seen on the right side of the picture (H&E; 40X)

**Fig 5:**
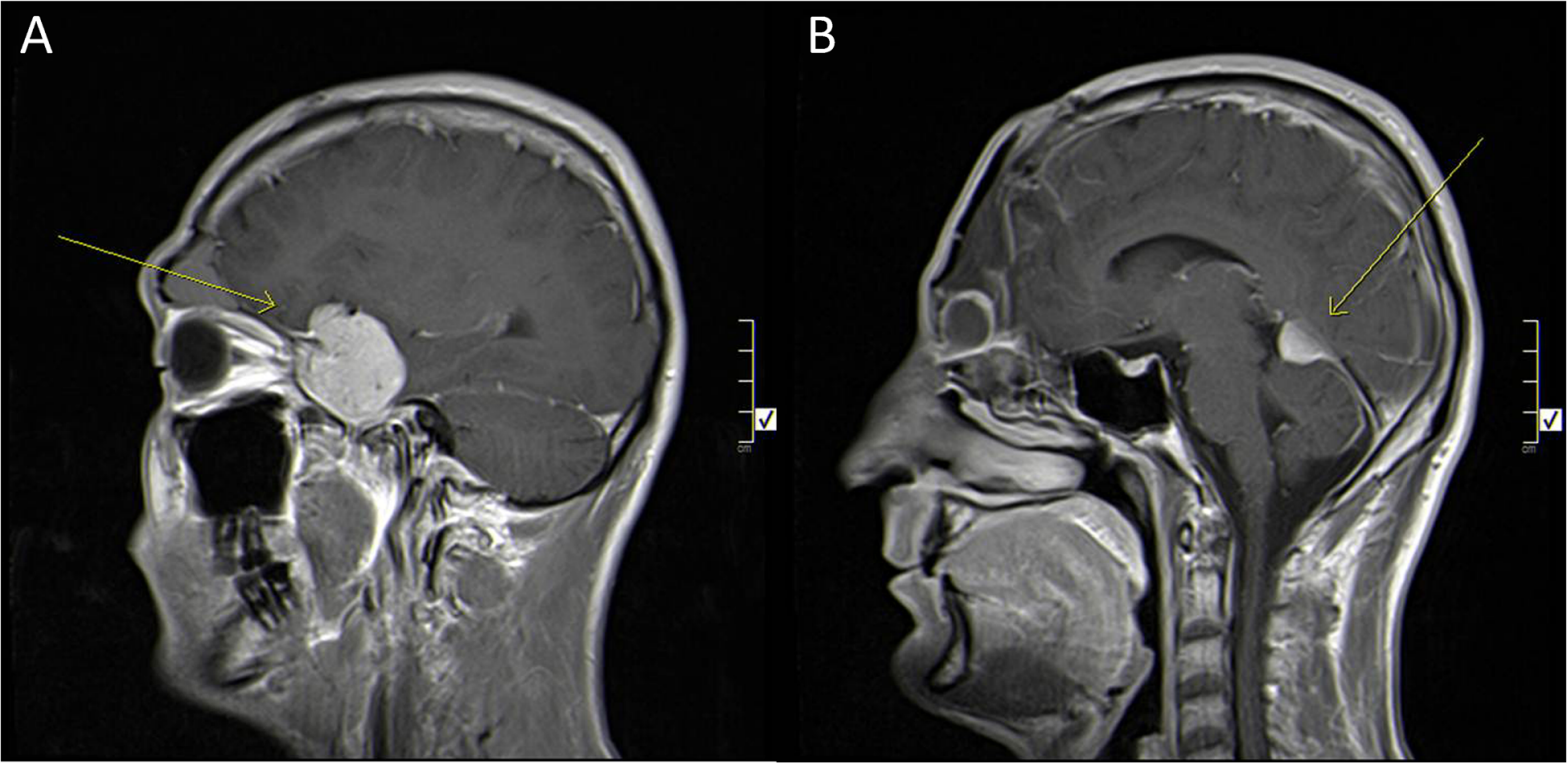
Brain MRI. Sagittal view. Post-contrast T1 weighted image. Showing **a**. recurrent mass in the infratentorial region, **b**. recurrent mass in the left temporal lobe

**Fig. 6:**
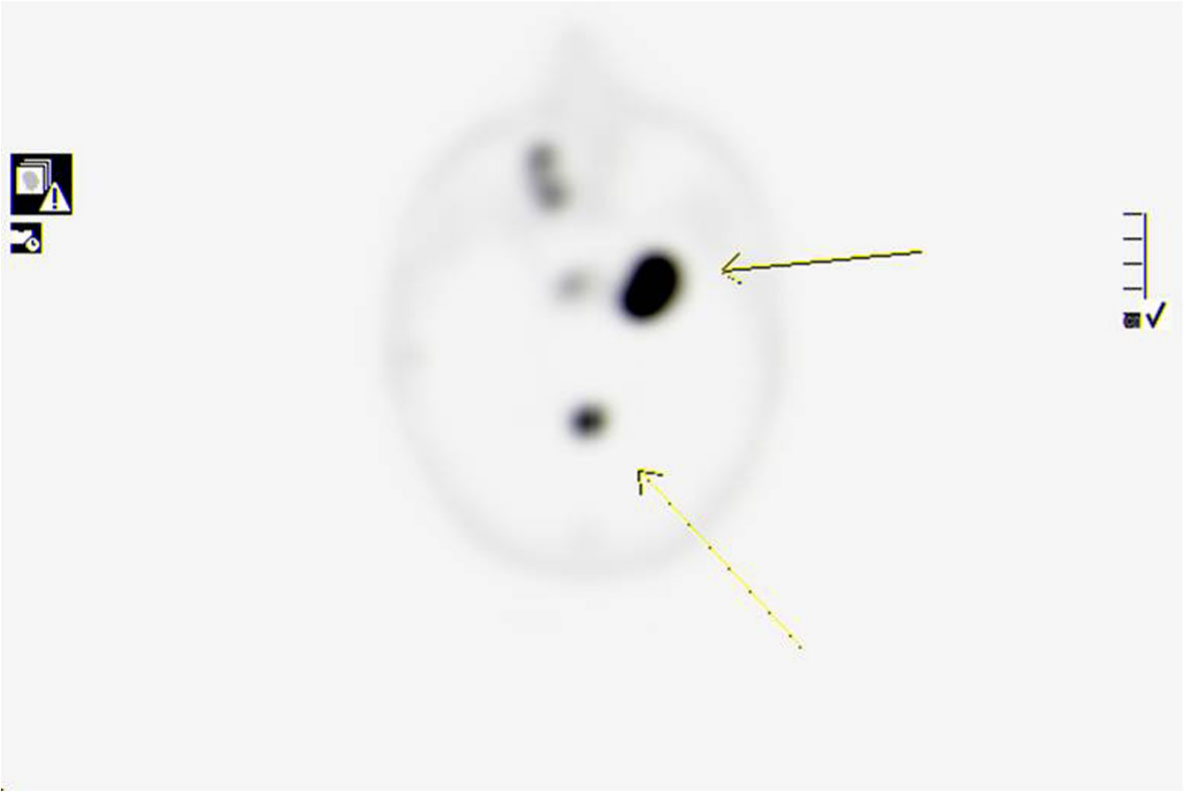
MIP reconstruction image of GA^68^ DOTATOC brain scan showing several somatostatin receptor positive lesions within the brain highly suspicious for metastatic process

On follow up chest CT scans, which took place in August of 2018, he was found to have new pleural based mass in the right lung apex, measuring about 3.3cm x 2.6 cm, there is progression in the size of the lingular pleural based nodule, now measuring about 1.5 cm, with stable tiny nodules in the right lung [Fig. 3]. After one month, he underwent left single-port VATS lower lobe wedge resection in which the thoracic cavity was approached from the 5^th^ intercostal space at the mid-axillary line, about 3.5 cm left upper lobe lingular mass not attached or invaded to the pleura was found and excised with safety margins and no pleural deposits were seen, and after two months it was followed by right single-port VATS upper lobe wedge resection and pleural mass resection, in which a 2 cm apical mass at right upper lobe, attached to apical mediastinal pleura, near to the sympathetic chain and brachial plexus, and the insertion of subclavian vein to superior vena cava (SVC). Another pleural nodule at the mid-SVC, about 6mm size and small scattered pleural nodules without effusion. Histopathology revealed metastatic paraganglioma in all masses except for the pleural nodule which was not a malignant [Fig. 4]. Patient has been on follow up ever since with a 3-month interval chest CT scan and a 6-months interval brain MRI. Unfortunately, a positron emission tomography (PET) scan using GA-68 DOTATOC and brain MRI at one year post metastasectomy showed local recurrence in primary lesion in the brain without any evidence of metastasis in the lungs or other organs [Figs. 5, 6] for which the patient is now receiving radiotherapy and the above mentioned recurrence is stable without any progression.

## Discussion

By definition, pheochromocytomas arise from adrenal medulla whereas paragangliomas arise from extra-adrenal paraganglia with an annual incidence of 1 in 300,000 for them combined [7–9]. Extra-adrenal paragangliomas are rare, highly vascular, non-epithelial tumors originating from neural crest-derived paraganglion cells situated in the region of autonomic nervous system ganglia and accompanying nerves, accounting for about 0.06% of all paragangliomas [10]. The histology, widely dispersed specialized neural crest chromaffin cells that are associated with autonomic ganglia [11], is similar to pheochromocytoma. However, as a group, they had higher frequency of metastases when compared to pheochromocytoma [12].

Paraganglioma is divided into two groups: one from the parasympathetic system and one from the sympathetic system based on the clinical and biological behavior. Intracerebral location of paraganglioma is rare, with cases being reported in the sella, cerebellopontine angle, penial body, frontal skull base, petrous ridge, sylvian fissure and cerebellum [10]. The development of paraganglioma in unusual regions such as the sella turcica might be due to the presence of remnants of paraganglionic tissue or due to abnormal migration; it is important to notice that no paraganglionic cells are detected in pituitary or adjacent tissue in adults. However, in fetal and neonatal period, neural crest tissue might be seen in avian embryo and these cells could be the origin of paraganglionic tissue. Furthermore, aggregates of paraganglionic cells were seen in glossopharyngeal nerve within petrous bone in previous reports [13].

Reithmeier T et al. reported a case of 42-year-old male patient with a history of vertigo and a single generalized seizure and was found to have an irregular contrast medium enhancing pathological structure within the sylvian fissure extending into the brain and he underwent complete resection using the frameless neuro-navigation and microsurgical technique [14]. Smith WT et al reported a case of a 17 year old female patient who had a left posterior fossa Chemodectoma (carotid body paraganglioma) who underwent complete resection via parietal flap which was followed up for 11 years 8 months without any signs of recurrence [15].

Paragangliomas are further differentiated based on their biochemistry into secreting (adrenergic,noradrenergic, or mixed phenotype) and non-secreting (biochemically silent) tumors. The latter most commonly arise from parasympathetic tissue in the head and neck area , such as the case we are presenting [16].

When metastasis are found, paragangliomas are considered to be malignant despite their well-known benign nature, with cells spreading via the lymphatic as well as the hematogenic route to local or distant lymph nodes and to other organs, such as the liver, lungs, and bones in order of decreasing frequency [17]. In the case we are presenting, we mentioned that the seen nodules increased in size from 1 cm to 1.5 cm on observation with the appearance of new nodules that were not seen on previous images. All of that solidified our decision that the seen nodules are of metastatic origin.

The slow recurrence rate of Paraganglioma necessitates long term follow up after surgical excision, that is considered the primary treatment option as reported by Lee et al, who reviewed 59 patients of the national cancer data base report with head and neck paragangliomas, and he also demonstrated that radiotherapy after surgery resulted in prolonged patient survival and slower tumor growth compared with patients not treated with adjuvant radiotherapy [18–20]. The introduction of somatostatin (SST)-receptor imaging agents (e.g. 68GaDOTA-TOC, 68Ga-DOTA-TATE) and combined functional/anatomical imaging (PET/CT) led to a dramatic improvement in detecting head and neck paragangliomas (HNPGL) and comparing radioisotopes 123I-MIBG and 18F-DOPA to this SST-analogue, 68Ga-DOTA-TOC appears to be the best choice in the detection of both, malignant and non-malignant HNPGL [21–26].

Managmenet of malignant paraganglioma includes a combination of surgical debulking, medical management in case of catecholamine excess, radionucleotide therapy (131I-MIBG or somatostatin analogues), chemotherapy (cyclophosphamide, vincristine, dacarbazine combination) [27], and external beam radiation therapy [28]. Keeping in mind that all of the above measures are considered palliative.

## Conclusion

Non-functional brain paraganglioma has an insidious onset without any typical symptoms, except for those caused by the mass compressing the adjacent structures. Treatment options are limited to surgical resection, chmoradiotherapy, or targeted therapy. Once metastases are diagnosed, PGL becomes a malignant disease with most therapeutic methods aimed to palliative measures.

Based on our literature search, this is the first case of non-functional frontal brain metastatic paraganglioma to the lungs, in which our patient underwent debulking surgery of the primary tumor followed by radiotherapy and after ensuring the disease stability, he had bilateral staged VATS metstasectomies.

## Data Availability

Not applicable.

## Abbreviations

PGL: Paraganglioma
VATS: Video-assisted thoracoscopic surgery
MRI: Magnetic resonance imaging
MRA: Magnetic resonance angiogram
ACA: Anterior cerebral artery
CT: Computed tomography
KHCC: King Hussein Cancer Center
SVC: Superior vena cava
PET: Positron emission tomography
SST: Somatostatin
HNPGL: Head and neck paraganglioma
